# Supplementary material for: N, S Co-Coordinated Zinc Single-Atom Catalysts for N-Alkylation of Aromatic Amines with Alcohols: The Role of S-Doping in the Reaction
Source: Nanomaterials (Basel). 2023 Jan 21;13(3):445. doi: 10.3390/nano13030445 (PMC9919690; doi:10.3390/nano13030445)
Supplement: Supplementary file 1 [file nanomaterials-13-00445-s001.zip › nanomaterials-2174949-supplementary.pdf]

Supporting Information

1. General Information

All chemicals are analytical grade and can be used without further purification. GC-MS was performed on an ISQ Trace 1300 in the electron ionization (EI) mode. GC analyses are performed on an Agilent 7890A instrument (Column: Agilent 19091J-413: 30 m × 320μm × 0.25μm, carrier gas: H_2, FID detection). ^{1}H NMR spectra is recorded on an AVANCE III Bruker spectrometer operating at 500 MHz in DMSO-d_6, respectively, and chemical shifts were reported in ppm relative to the center of the singlet at 2.50 ppm for DMSO-d_6.

2. Experimental section

2.1. Catalysts preparation

Synthesis of CN@Zn_1-AA. Firstly, 2-methylimidazole (24 mmol), aniline (24 mmol) and ammonia (3 mL) were added to the zirconia ball mill, and the homogeneous solution A was obtained by dissolving it with ethanol (16 mL). Zinc nitrate hexahydrate (6 mmol) was dissolved in deionized water (5 mL) to obtain zinc nitrate aqueous solution B. Solution B was poured into solution A, and then ball-milling was carried out at 400 rpm for 2 h. The resulting viscous white liquid is centrifuged, washed with deionized water several times, and then dried at 80 °C overnight in a vacuum oven to obtain the precursor. Then, the prepared precursor was calcined at 900 °C for 2 h in a tube furnace with a ramping rate of 5 °C/min in nitrogen atmosphere to obtain CN@Zn_1-AA.

Synthesis of CNS@Zn_1, CNS@Zn_1-AA, CNS@Zn_1-Am, CNS@Zn_1-An. All of these N-doped carbon supported Zn catalystswere prepared by the similar procedure as CN@Zn_1-AA except for the type and ratio of additives.

In the cases of CNS@Zn_1, CNS@Zn_1-AA, CNS@Zn_1-Am, CNS@Zn_1-An, 6 mmol thiourea (molar ratio of S/Zn = 1) was added to solution A. In the case of CNS@Zn_1, only 2-methylimidazole was added to solution A. In the case of CNS@Zn_1-AA, 2-methylimidazole, aniline and ammonia were added to solution A. In the case of CNS@Zn_1-Am, only 2-

methylimidazole and ammonia were added to solution A. In the case of CNS@Zn_1-An, only 2-methylimidazole and aniline were added to solution A.

2.2. Characterization

XRD analysis is performed on Shimadzu X-ray diffractometer (XRD-6000) with Cu Kα irradiation. Transmission electron microscopy (TEM) images are taken using a PHILIPS Tecnai 12 microscope operating at 120 kv. Scanning electron microscopy (SEM) images are performed using a Hitachi S-4800 apparatus on a sample powder previously dried and sputter -coated with a thin layer of gold. Atomic-resolution HAADF-STEM images are taken using a FEI Titan Cubed Themis G2 300 S/TEM with a probe corrector and a monochromator at 200 kV. X-ray photoelectron spectroscopy (XPS) is performed on an ESCALAB 250Xi spectrometer, using an Al Kα X-ray source (1350 eV of photons) and calibrated by setting the C 1s peak to 284 .80 eV. Inductively coupled plasma mass spectrometry (ICP-MS) is analyzed on Optima 7300 DV. BET surface area and pore size measurements are performed with N_2 adsorption/desorption isotherms at 77 K on a Micromeritics ASAP 2020 instrument. Before measure ments, the samples are degassed at 150 °C for 12 h. The X-ray absorption fine structure spectras (Zn K-edge) are collected at BL14W beamline in Shanghai Synchrotron Radiation Facility (SSRF). The storage rings of SSRF is operated at 3.5 GeV with a stable current of 200 mA. Using Si(111) double-crystal monochromator, the data collection were carried out in fluorescence mode using Lytle detector. All spectra are collected in ambient conditions. Data reduction, data analysis, and EXAFS fitting are performed with the Athena and Artemis software packages. The energy calibration of the sample is conducted through a standard Zn foil, which as a reference is simultaneously measured. For EXAFS modeling, EXAFS of the Zn foil is fitted and the obtained amplitude reduction factor S02 value (0.700) is set in the EXAFS analysis to determine the coordination numbers (CNs) in the Zn-N/S scattering path in sample.

2.3. Catalytic reaction

In a typical reaction procedure, 1 mmol aniline, 2 mmol benzyl alcohol, 0.3 mmol KOH, 2 mL toluene and 15 mg CNS@Zn_1-AA (0.9 mol% Zn) were placed in a 25 ml sealed tube, and then the tube was purged with argon gas. The reaction mixture was heated to 120 °C for 12 h. After the

reaction was completed, the sealed tube was cooled down to room temperature, and the solution was diluted with ethyl acetate, and analyzed by gas chromatography (GC) and GC-mass spectrometry (GC-MS). The corresponding product was purified by column chromatography with silica gel (ethyl acetate/ petroleum ether).

2.4. Recyclability of CNS@Zn_1-AA

After the reaction was completed, the catalyst was separated by centrifugation, washed with ethyl acetate and dried, and directly reused for the next reaction cycle.

3. Computational details

Spin-unrestricted density functional theory (DFT) calculations were conducted in the Dmol3 module (BIOVIA Inc.) [1] using a generalized gradient approximation (GGA) with the Perdew-Burke-Ernzerhof (PBE) exchange correlation functional [2]. Double numerical basis set with polarization (DNP) was used in the calculations, which was reliable to describe the valence orbital of the atoms here. A Fermi smearing value of 0.005 Hartree was also used to improve computational performance. The convergence criteria for the energy, force and displacement were set to 10-5 Hartree, 0.002 Hartree/Å and 0.005 A, respectively. To avoid periodic interactions, a 4×4×1 supercell with a vacuum space of 20.0 Å was used to simulate the adsorption. The transition states were located using the complete linear synchronous transit/quadratic synchronous transit (LST/QST) method. And the frequencies of them are examined to conform the validity [3].

Reference

1. Delley, B. From molecules to solids with the $DMol^3$ approach. *J. Chem. Phys.* **2000**, 113, 7756-7764.

2. Perdew, J. P.; Burke, K.; Ernzerhof, M. Generalized gradient approximation made simple. *Phys. Rev. Lett.* **1996**, 77, 3865-3868.

3. Halgren, T. A.; Lipscomb, W. N. The synchronous-transit method for determining reaction pathways and locating molecular transition states. *Chem. Phys. Lett.* **1977**, 49, 225-232.

4. Results and discussion

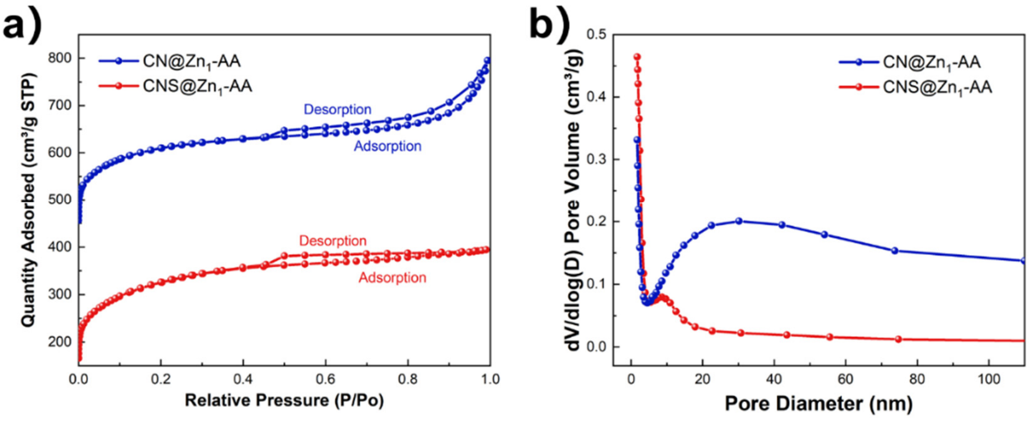

Figure S1 CN@Zn1-AA and CNS@Zn1-AA images of (a) Brunauer-Emmett-Teller (BET); (b) Barrett-Joyner-Halenda (BJH).

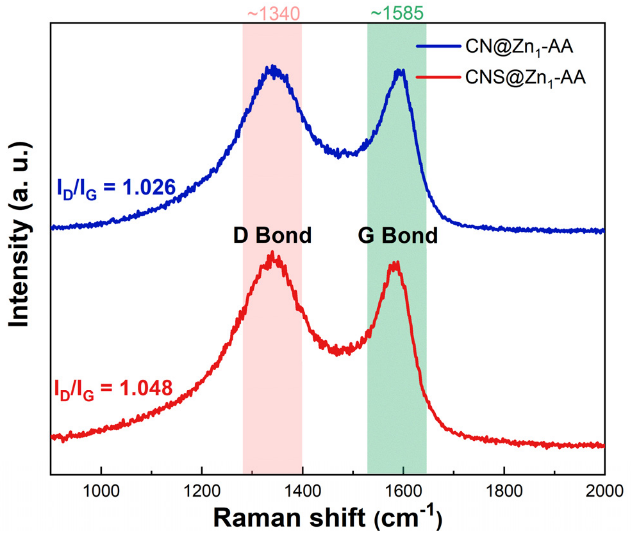

Figure S2 The Raman spectra of CN@Zn_1-AA (blue) and CNS@Zn_1-AA (red).

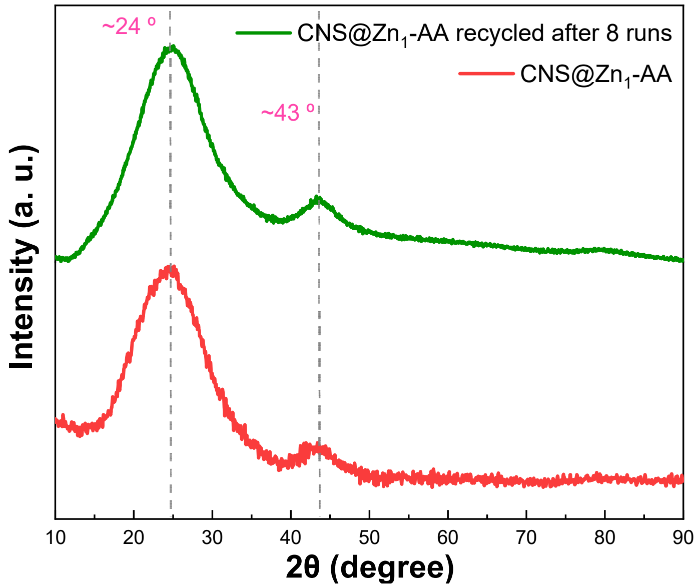

Figure S3 XRD image of CNS@Zn_1-AA (red) and recycled CNS@Zn_1-AA after 8 runs (green).

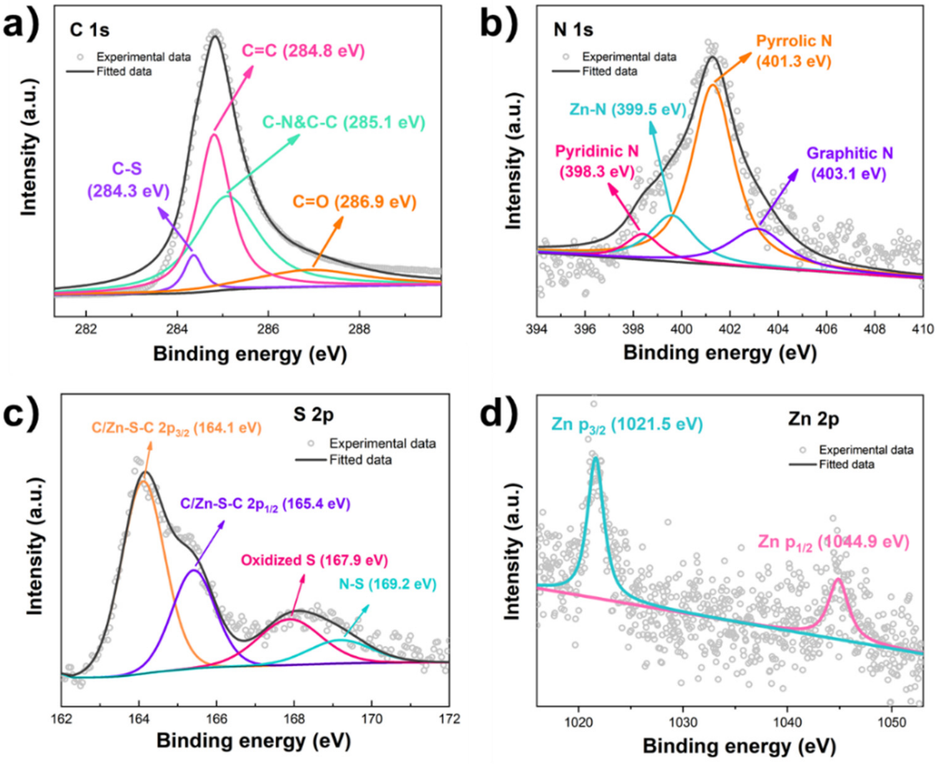

Figure S4 XPS spectra of CNS@Zn_1-AA.

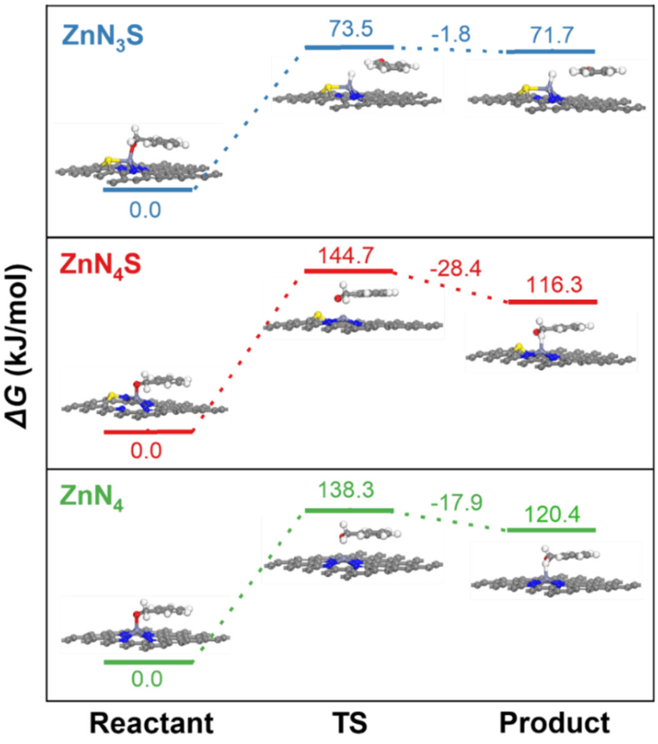

Figure S5 Partial reaction pathway and corresponding energies of ZnN_3S, ZnN_4S and ZnN_4 for the N-alkylation of amines with benzyl alcohol.

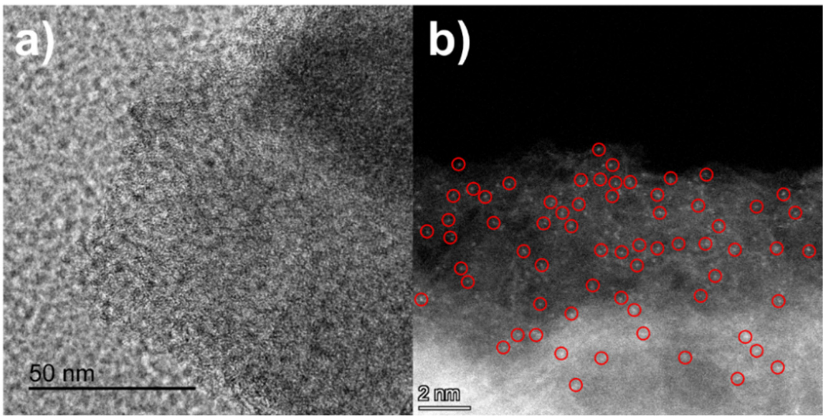

Figure S6 (a) TEM and (b) HAADF-STEM images of CNS@Zn_1-AA recycled after 8 runs.

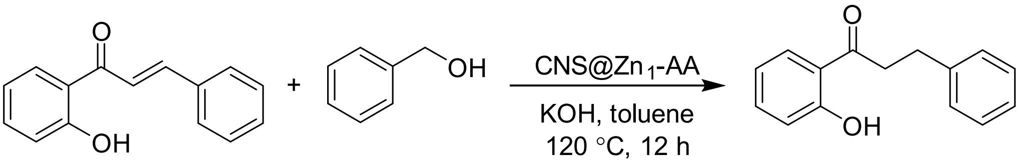

Scheme S1 Reduction of o-hydroxychalcone with benzyl alcohol. Reaction conditions: **5** (1 mmol), **2a** (2 mmol), KOH (0.3 eq.), CNS@Zn_1-AA (15 mg), Ar, 120 ˚C, 12 h.

Table S1 The BET surface areas and pore volumes of CN@Zn_1-AA and CNS@Zn_1-AA.

Catalyst	BET Surface Area	Average pore diameter (4V/A by BET)
CNS@Zn_1-AA	1080 m^2/g	2.09 nm
CN@Zn_1-AA	964 m^2/g	2.86 nm

Table S2 ICP result of CNS@Zn1-AA.

Catalyst	Element	Zn content (mg/kg)
CNS@Zn1-AA	Zn	38826

Table S3 K-edge EXAFS fitting parameters of CNS@Zn1-AA catalyst (S_0^2=0.700).

Sample	Shell	CN[a]	R(Å)[b]	σ^2(Å^2)[c]	ΔE_0(eV)[d]	R factor
Zn foil	Zn-Zn	12*	2.64±0.01	0.0138±0.0011	-2.7±1.0	0.0116
Zn	Zn-N	3.7±0.7	2.02±0.02	0.0059±0.0050	3.5±1.2	0.0091
	Zn-S	1.0±0.5	2.28±0.03	0.0059±0.0050		

[a] CN, coordination number. [b] R, distance between absorber and backscatter atoms. [c] σ^2, Debye-Waller factor to account for both thermal and structural disorders. [d] ΔE_0, inner potential correction; R factor indicates the goodness of the fit. S_0^2 was fixed to 0.700, according to the experimental EXAFS fit of Zn foil by fixing CN as the known crystallographic value. Fitting range: $3.0 \leq k$ (/Å) ≤ 12.6 and $1.5 \leq R$ (Å) ≤ 3.0 (Zn foil); $2.0 \leq k$ (/Å) ≤ 10.0 and $1.1 \leq R$ (Å) ≤ 3.05 (Zn). A reasonable range of EXAFS fitting parameters: $0.700 < S_0^2 < 1.000$; CN > 0; $\sigma^2 > 0$ Å^2; $\Delta E_0 < 10$ eV; R factor < 0.02.

5. ^{1}H NMR spectra of selected products

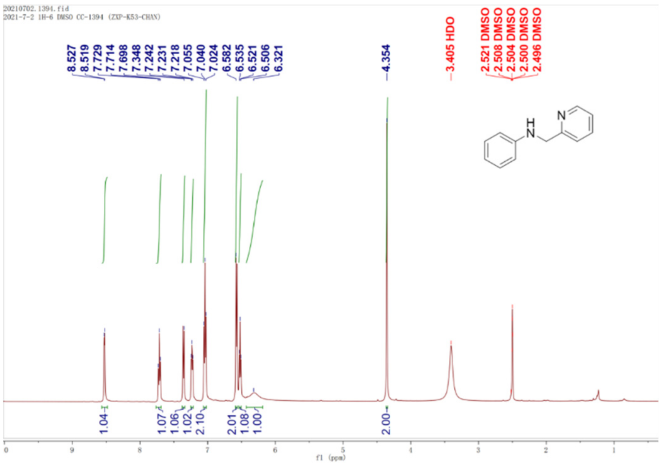

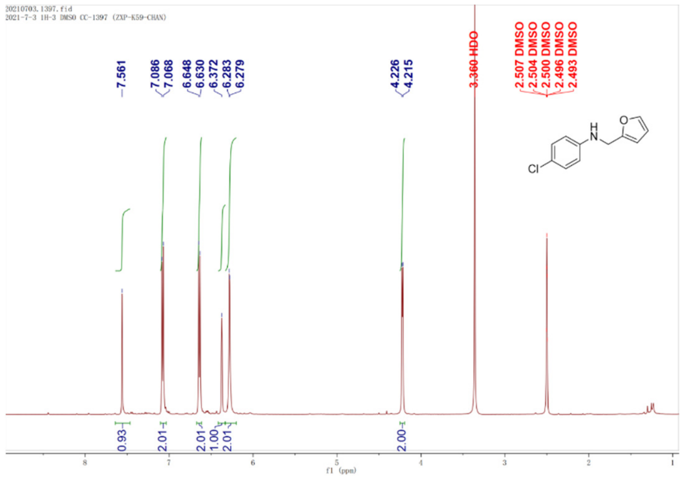
20210703.1397.fid
2021-7-3 1H-3 DMSO CC-1397 (ZXP-K59-CHAN)
7.561
7.086
7.068
6.648
6.630
6.372
6.283
6.279
4.226
4.215
3.360 HDO
2.507 DMSO
2.504 DMSO
2.500 DMSO
2.496 DMSO
2.493 DMSO
Cl
H
N
O
0.93
2.01
2.01
1.00
2.01
2.00
f1 (ppm)

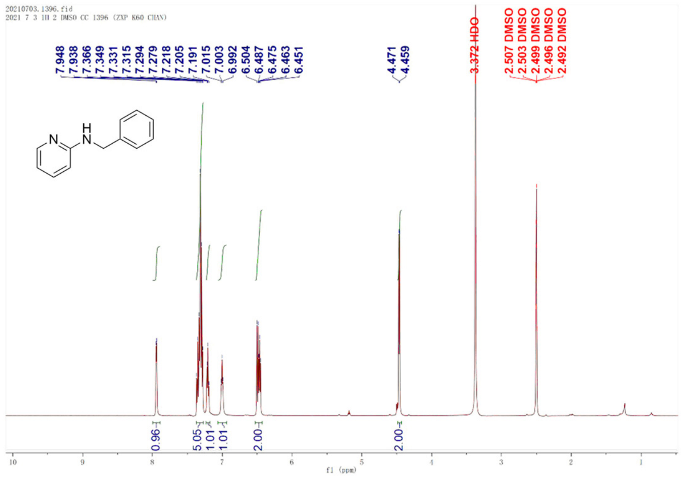
20210703.1396.fid
2021 7 3 1H 2 DMSO CC 1396 (ZXP K60 CHAN)
7.948
7.938
7.366
7.349
7.331
7.315
7.294
7.279
7.218
7.205
7.191
7.015
7.003
6.992
6.504
6.487
6.475
6.463
6.451
4.471
4.459
3.372 HDO
2.507 DMSO
2.503 DMSO
2.499 DMSO
2.496 DMSO
2.492 DMSO
N
H
N
0.96
5.05
1.01
1.01
2.00
2.00
f1 (ppm)

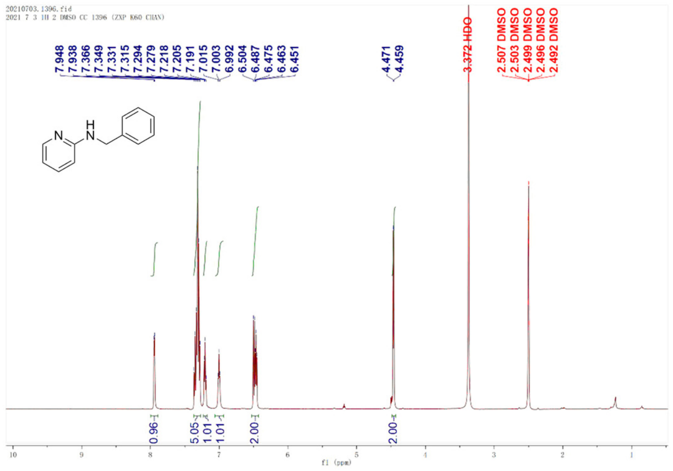
20210703.1396.fid
2021 7 3 1H 2 DMSO CC 1396 (ZXP K60 CHAN)
7.948
7.938
7.366
7.349
7.331
7.315
7.294
7.279
7.218
7.205
7.191
7.015
7.003
6.992
6.504
6.487
6.475
6.463
6.451
4.471
4.459
3.372 HDO
2.507 DMSO
2.503 DMSO
2.499 DMSO
2.496 DMSO
2.492 DMSO
N
H
N
0.96
5.05
1.01
1.01
2.00
2.00
f1 (ppm)

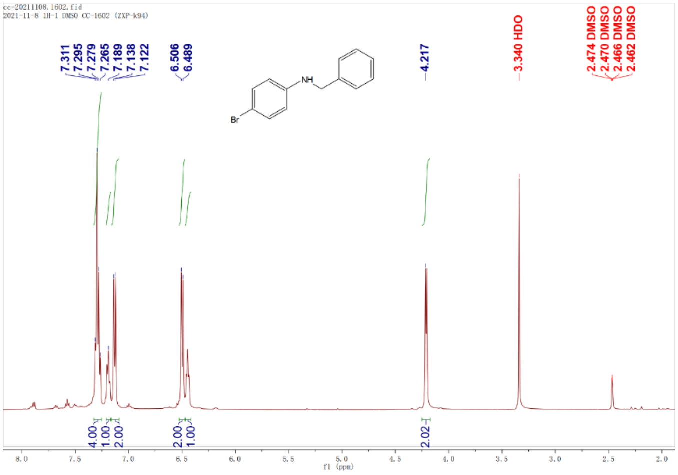
cc-20211108.1602.fid
2021-11-8 1H-1 DMSO CC-1602 (ZXP-k94)
7.311
7.295
7.279
7.265
7.189
7.138
7.122
6.506
6.489
4.217
3.340 HDO
2.474 DMSO
2.470 DMSO
2.466 DMSO
2.462 DMSO
NH
Br
4.00
1.00
2.00
2.00
1.00
2.02
f1 (ppm)
